# Intermolecular Interactions between Aldehydes and Alcohols: Conformational Equilibrium and Rotational Spectra of Acrolein-Methanol Complex

**DOI:** 10.3390/molecules29153444

**Published:** 2024-07-23

**Authors:** Dingding Lv, David Sundelin, Assimo Maris, Luca Evangelisti, Wolf Dietrich Geppert, Sonia Melandri

**Affiliations:** 1 Dipartimento di Chimica “G. Ciamician” Università di Bologna, Via Selmi 2, I-40126 Bologna, Italy; 2 Fysikum, Stockholm University, Roslagstullsbacken 21, 106 91 Stockholm, Sweden; david.sundelin@fysik.su.se

**Keywords:** acrolein, methanol, hydrogen bond, microwave spectroscopy, molecular structure, methyl internal rotation

## Abstract

The rotational spectra of the 1:1 complex formed by acrolein and methanol and its deuterated isotopologues have been analyzed. Two stable conformations in which two hydrogen bonds between the two moieties are formed were detected. The rotational lines show a hyperfine structure due to the methyl group internal rotation in the complex and the *V_3_* barriers hindering the motion were determined as 2.629(5) kJ mol^−1^ and 2.722(5) kJ mol^−1^ for the two conformations, respectively. Quantum mechanical calculations at the MP2/aug-cc-pVTZ level and comprehensive analysis of the intermolecular interactions, utilizing NCI and SAPT approaches, highlight the driving forces of the interactions and allow the determination of the binding energies of complex formation.

## 1. Introduction

Hydrogen bonds (HBs) have long been of paramount interest to the chemical community. Some types of molecules (for example, alcohols [1], acids [2] and amides [3]) are very prone to form HBs, both as donors and acceptors. The conformational preference in forming HBs affects the energetics and kinetics of chemical reactions and the structure of biological macromolecules [4,5]. Complexes involving aldehydes and alcohols are particularly interesting systems in HB studies because they often contain a combination of stronger HBs and weaker secondary interactions. This study of clusters of aldehydes and alcohols aims to determine the interaction sites and relative arrangement of monomers and determine the intermolecular forces at play, and it lays the foundation for studies of larger systems.

In the past few decades, the scientific community has made great progress in the understanding of the nature and properties of the hydrogen bond. Hydrogen-bonded clusters have enjoyed considerable attention from the scientific community due to their importance in many fields of chemistry, e.g., in atmospheric and biological processes. They also can serve as simple model systems to aid the understanding of the structures and intermolecular interactions in bulk solutions. In this respect, clusters by water and simple organic molecules like alcohols, which are often used as solvents in synthetic processes (both in the lab and industries), play an eminent role. The cluster bonding between water and alcohols with organic molecules can be very strong. This especially holds for species in which the water or the alcohol can act both as a hydrogen acceptor and a hydrogen donor, like in the case of 2-aminopyridine-water [6]. The formation of clusters of organic compounds with small molecules can also change the geometry of the species. For example, it was found that the clustering of benzaldehyde with a larger number of water molecules distorts the planar structure of the benzaldehyde moiety [7]. Very recently, a similar behavior was detected for water clusters of salicylic acid [8]. Also, in crown ethers, a change in the conformation of the organic molecule upon clustering with water was observed [9]. Very often, several isomers with similar energies exist for organic water clusters, so high-level ab initio calculations combined with rotational spectroscopy are necessary to pin down the correct geometries of the clusters [10]. In a number of instances, more than one isomer has been observed, as in the cases of 2- and 3-furonitrile [11], and for the formamide-water cluster, even three different isomers could be detected [12]. Furthermore, the clustering of organic molecules, especially α-unsaturated aldehydes like acroleine, can affect their reactivity and the product distributions of the reactions they undergo [13,14,15]. Since acrolein is a major pollutant in the terrestrial atmosphere, this is of high relevance.

Moving from water to methanol clusters increases the complexity of the spectra by introducing the possibility of internal rotation of the methyl group. This phenomenon and the associated rotational barrier have since long been a subject of great attention from the spectroscopic community. For example, the cluster of thioacetaldehyde was investigated by Judge et al. already in 1987 [16]. Clustering of methanol tends to lower the internal rotational barrier. Earlier, this was attributed to the complexation, but more recent research efforts have interpreted it as being due to an internal motion coupled to the internal rotation of the methyl group, namely a librational motion of the entire MeOH moiety, the methyl group or even the hydroxyl group [17,18]. Nevertheless, further studies of different methanol clusters are necessary to fully understand this phenomenon, since such endeavors have implications for understanding the stability and interactions of complexes involving aldehydes and alcohols, contributing to the broader field of chemical and biological molecular research. Expanding the number of systems studied also makes it possible to identify the influence of changes in the nature of substituents on the nature and energy of non-covalent binding and the existence of various conformers of adducts.

Acrolein (from here on ACR) is also one of the most important environmental pollutants and plays an important role in the formation of atmospheric aerosols [19,20,21]. It is also a very important synthetic intermediate in the chemical industry and is widely used in resin production and organic synthesis [22]. Two conformations of ACR, s-*trans* and s-*cis*, have been characterized by microwave spectroscopy [23,24,25,26,27,28,29], pointing out that the s-*trans* form is more stable than the s-*cis* one by 9.2(5) kJ mol^−1^ [30]. The presence of those two different conformers of the ACR monomer can make the investigation of the complex formation more interesting, since the relative energy of the conformations can change upon complexation. The rotational spectra of ACR involved in binary and ternary water complexes and some of their isotopologues have been analyzed [31]. All the complexes show a planar heavy atom skeleton and are stabilized by ring-like structures. In the binary complex ACR-water [31], the conformation where the aldehyde oxygen forms an HB with the water hydrogen and the water oxygen forms an HB with the methylene group are most stable and the ACR adopts the s-*trans* form. The electron pairs located at the aldehyde oxygen are quite efficient proton acceptors that can easily form an HB with proton donors, like water, alcohols or other protonated compounds. At the same time, the oxygen atom of methanol (from here on abbreviated as MeOH) can easily form another interaction with the methylene hydrogen or aldehydic hydrogen atoms of ACR. In this case, it is possible that the 1:1 complex of ACR and MeOH will also show a ring-like structure where aldehyde oxygen interacts with hydroxyl hydrogen and hydroxyl oxygen interacts with methylenic hydrogen or aldehydic hydrogen.

The rotational spectra of the MeOH monomer and those of many of its clusters with other molecules have been studied [17,18,21,32,33,34,35,36,37,38,39,40,41,42,43,44]. The splitting of the rotational lines caused by the internal rotation of the methyl group was always observed and the determined barriers of the methyl group’s internal rotation in the complexes were lower than the one determined for the MeOH monomer (4.463(4) kJ mol^−1^ [44]). For example, in the 1:1 complex of MeOH-SO_2_ [37], the observed methyl top internal rotation barrier is *V*_3_ = 1.54(1) kJ mol^−1^ and in the more loosely bound MeOH-Ar complex [39], an even more striking drop in the methyl torsional barrier height to about 0.82(2) kJ mol^−1^ was observed in the experiment. The model used to deduce the barrier height is based on the assumption that the methyl group rotates against a heavy frame, but the experimental data confirm that the barrier reductions are not a result of complexation effects, but rather are related to another large amplitude internal motion coupled to the internal rotation of the methyl group. In the note by Fraser et al. [17], the decrease in the MeOH internal rotation barrier observed in several methanol complexes with light partners is attributed to the effects of a librational motion either of the whole MeOH moiety or of the methyl group taking place in the complex. This might not be the case when a heavier moiety is complexed by MeOH. For example, in a more recent study of the 1:1 complex of acrylonitrile−MeOH in the gas phase using rotational spectroscopy [18], a consistent change was not observed in the determined internal rotation parameters upon deuteration of the whole methyl group, which led the authors to exclude that the decrease in the MeOH internal rotation barrier after complexation is related to the coupling of another internal motion of the whole MeOH moiety or the methyl group. 

The study of the driving forces stabilizing the molecular complexes and the effects of complexation on the structure and the internal motions are the aims of this study. 

Rotational spectroscopy provides high resolution and exceptional sensitivity to the molecular mass distribution and geometry, making it ideal for obtaining precise experimental evidence of tautomeric equilibria. In the present study, a COBRA-type pulsed supersonic-jet) spectrometer was employed to measure the radiofrequency spectrum of the ACR-methanol cluster. Deuterated isotopologues of methanol were used to validate the assignment of spectra, to assess the effects of deuteration on the A-E line splitting and the influence of deuteration upon the methyl top internal rotation barrier of the cluster and to compare those parameters with previously studied systems. The high spectral resolution of state-of-the-art Fourier transform microwave spectrometers allows the full resolution of closely spaced rotational spectra of isotopically labeled clusters. Ab initio at the MP2/aug-cc-pVTZ were employed to identify the structures of the different clusters and to determine their relative energies. Moreover, such calculations yield rotational constants and electric dipole moment components of the different conformations, which can aid the assignment of lines, identify experimentally observed isomers, and be used as benchmarks for the experimentally found parameters (and vice versa). The combination of rotational spectroscopy analysis and theoretical methods thus provides a synergistic method for studying the structure and internal dynamics of isolated molecules and weakly bound complexes.

## 2. Results and Discussion

### 2.1. Computational Results

For simplicity, the s-*trans* and s-*cis* conformations of ACR are labeled just *t* and *c*. Distinct conformers of ACR-MeOH, which originate from the same conformer of ACR have been further distinguished with a progressive number (1, 2 or 3). The choice of the initial geometries was based on the previous study on ACR-water [31]. In that case, four planar conformers, in which the water forms an HB with the ACR oxygen atom and a secondary interaction involving the water oxygen with either the methylenic or the aldehydic ACR hydrogen, were identified. In the ACR-MeOH case, the same kind of C_s_ arrangement is found, where the methanol frame lies in the plane of acrolein, but also, two additional non-planar conformers (one from each of ACR’s conformation and indicated by the number 3) were characterized. In both cases, the methyl group is located almost perpendicular to the ACR’s plane. The theoretical (MP2/aug-cc-pVTZ) spectroscopic parameters and relative energies of all conformers are reported in Table 1. 

The complexes of MeOH with *t*-ACR show, in general, lower energies than those with *c*-ACR. In fact, the three lowest energy structures are the complexes of MeOH with *t*-ACR and span about 3.7 kJ mol^−1^, and the lowest energy structure formed by *c*-ACR (*c*-ACR-MeOH-1) is located 10.5 kJ mol^−1^ higher. This energy difference is very close to the energy difference (9.2(5) kJ mol^−1^) determined for the two forms of the ACR monomer [30] and the energy difference (9.9 kJ mol^−1^) for the *t*- and *c*-ACR complexes with water [31]. 

The six lowest energy structures (three originating from *t*-ACR and three from *c*-ACR) all exhibit a strong OH···O HB and a weak C–H···O interaction. The hydroxyl group of MeOH acts as a proton donor for the OH···O interaction and as a proton acceptor for the C–H···O interaction. The conformations 1 and 3, in which the MeOH oxygen forms a weak interaction with the methylenic hydrogen, are more stable than the conformations labeled 2 where the oxygen is bound to the aldehydic hydrogen. For example, the conformer *t*-ACR-MeOH-2 is 3.7 kJ mol^−1^ higher in energy than *t*-ACR-MeOH-1 and the conformer *c*-ACR-MeOH-2 is 3.0 kJ mol^−1^ higher in energy than *c*-ACR-MeOH-1.

Considering conformations 1 and 3, where the MeOH oxygen is bound to the methylenic hydrogen, the planar conformers show lower energy than the non-planar ones, although, in general, those differences are smaller than the ones between 1 and 2. The energy difference between *t*-ACR-MeOH-3 and *t*-ACR-MeOH-1 is 1.4 kJ mol^−1^, and the energy difference between *c*-ACR-MeOH-3 and *c*-ACR-MeOH-1 is 0.5 kJ mol^−1^. 

### 2.2. Spectroscopic Results

According to the computational results, *t*-ACR-MeOH-1 is the global minimum, and it shows the most intense spectrum originating from the *μ*_a_ electric dipole moment component, which is much higher in value with respect to *μ*_b_ and *μ*_c_ (the latter one being zero by symmetry). We started our experiment with the search for the *J*′ ←*J*″: 3←2 *μ*_a_-R rotational transition of *t*-ACR-MeOH-1, and after the detection and fit of one group of rotational transitions, the measurements were extended following new predictions to 42 rotational transitions with the rotational quantum number *J* ranging from 1 to 6 and *K*_a_ ranging from 0 to 2, including *μ*_b_ type transition lines. 

After finding all detectable rotational transition lines of the conformer *t*-ACR-MeOH-1, the measurements were extended to detect those of conformer *t*-ACR-MeOH-2. From the calculated relative energy and considering the temperature prior to the expansion (273 K), the population of *t*-ACR-MeOH-2 was predicted to be 19.6% of the global minimum, and taking into account the values of the dipole moment components, the most intense *μ*_a_ lines of *t*-ACR-MeOH-2 were predicted to be 22.0% of the most intense ones of *t*-ACR-MeOH-1. Indeed, 42 rotational transitions with the rotational quantum number *J* ranging from 1 to 7 and *K*_a_ ranging from 0 to 2 were observed for *t*-ACR-MeOH-2, including *μ*_b_ type transition lines.

The list of frequencies for both conformers is reported in the Supporting Information (Tables S1–S4), while the spectral parameters of the observed conformations of *t*-ACR-MeOH-1 and *t*-ACR-MeOH-2 obtained from a direct fit of the lines through the XIAM program [45] are summarized in Table 2. The fitted rotational constants are within 3% of the ones predicted from theory for both conformers. The detected *μ*_a_-type and *μ*_b_-type rotational transitions and the non-observation of the *μ*_c_-type lines confirm the assignment to the planar conformers, which are in agreement with the zero value of the *μ*_c_ dipole moment components for both two conformers.

**Figure 1:**
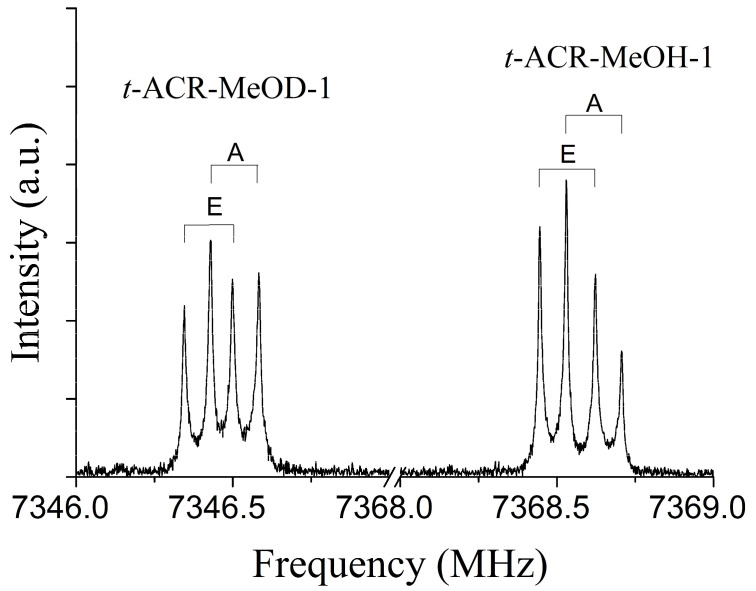
The 3_03_←2_02_ transitions of *t*-ACR-MeOH-1 and *t*-ACR-MeOD-1 enriched isotopologue showing the methyl internal rotation A-E doublets. Each transition appears as a doublet (indicated by square brackets) due to the instrumental Doppler effect.

As an example, the 3_03_←2_02_ transition for the *t*-ACR-MeOH-1 conformer is shown in Figure 1 where the splitting of the rotational lines due to the internal rotation of the methyl group of MeOH can be observed; A and E are methyl torsional state symmetry labels.

### 2.3. Determination of Spectroscopic Parameters and Torsion Barriers

From the fitting procedure performed on the experimental transition frequencies, in addition to the rotational constants (*A*, *B*, *C*) and the quartic centrifugal distortion constants (*D*_J_, *D*_JK_ and *d*_1_), internal rotation parameters are also obtained, as reported in Table 2. The determined internal rotation parameters are *V*_3_, the three-fold barrier to internal rotation of the methyl group, *F*_0_, the rotational constant of the internal rotor, and *δ*, the angle between the internal rotor axis and the *a*-principal axis of inertia, while *D*_c3J_ is an empirical internal-overall rotation distortion constant. The determined *V*_3_ is smaller in *t*-ACR-MeOH-1 (2.686(3) kJ mol^−1^) than in *t*-ACR-MeOH-2 (2.723(6) kJ mol^−1^), but both conformers have a low internal rotational barrier compared to the MeOH monomer (4.463(4) kJ mol^−1^ [44]).

**Figure 2:**
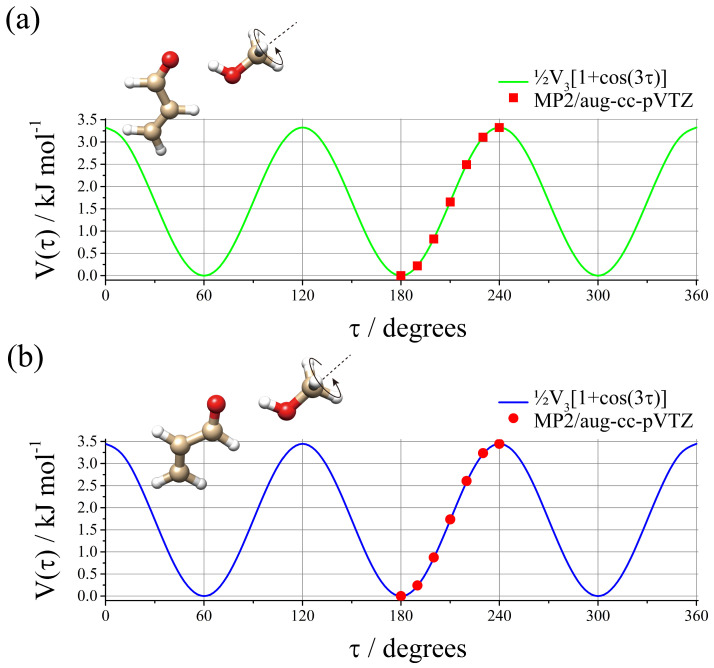
Ab initio (MP2/aug-cc-pVTZ) methyl internal rotational potential energy surface for (**a**) *t*-ACR-MeOH-1 and (**b**) *t*-ACR-MeOH-2.

In order to understand the reason for the lowering of the *V*_3_ barrier in the complex compared to the MeOH monomer, the potential energy surface of the MeOH methyl torsion was calculated by changing the dihedral angle of *τ* = HC-OH on the regular grid with Δ*τ* = 10°. The results are shown in Figure 2 and the calculated data, expressed in red bullets and dots, are well reproduced by the threefold function: *V*(*τ*) = ½*V*_3_[1 + cos(3*τ*)], which is shown as a green line for *t*-ACR-MeOH-1 and a blue line for *t*-ACR-MeOH-2. The maximum value of *t*-ACR-MeOH-1 (3.32 kJ mol^−1^) represents the theoretical barrier hindering the methyl group internal rotation in the complex, and it can be noted that it is larger (about 26%) than the experimental values obtained for the normal species, *V*_3_ = 2.686(3) kJ mol^−1^ for *t*-ACR-MeOH-1 (see Table 2). The difference between the theoretical (3.45 kJ mol^−1^) and experimental (2.723(6) kJ mol^−1^) *V*_3_ values was also observed in *t*-ACR-MeOH-2. It is possible to attribute these differences to the accuracy of the theoretical method. This can be tested by calculating the barrier to internal rotation for free MeOH with the same theoretical method (4.16 kJ mol^−1^) and comparing it to the experimental barrier (4.50 kJ mol^−1^) [46]. The calculated barrier for free MeOH is indeed lower than the experimental one but only by about 7%, which is much smaller than the 26% difference found for *t*-ACR-MeOH-1 and the 27% for *t*-ACR-MeOH-2. This difference could be due to a lower accuracy of the method in calculating methyl torsional barriers in molecular complexes or to a possible large amplitude motion coupled to the torsion for which no evidence could be found in the experimentally measured rotational spectrum. 

### 2.4. Deuterated Isotopologues

The rotational spectra of the deuterated isotope of methanol, abbreviated as *t*-ACR-MeOD-1 and *t*-ACR-MeOD-2, were also measured. Similarly, to the spectra of the parent species, the peaks of the deuterated isotopologues show up as doublets due to the internal rotation of the methyl group. The spectral parameters obtained with the same fitting procedure used for the undeuterated species are summarized in Table 2, and the observed transition frequencies of all measured isotopes are provided in the Supporting Information (Tables S3 and S4). As shown in Table 2, the deuterated species *t*-ACR-MeOD-1 (*V*_3_ = 2.825(3) kJ mol^−1^) and *t*-ACR-MeOD-2 (*V*_3_ = 2.856(1f) kJ mol^−1^) shows a higher barrier of *V*_3_ than the corresponding parent species *t*-ACR-MeOH-1 (*V*_3_ = 2.686(3) kJ mol^−1^) and *t*-ACR-MeOH-2 (*V*_3_ = 2.723(6) kJ mol^−1^). One would therefore expect to find a larger A–E splitting of the spectrum of the deuterated species *t*-ACR-MeOD-1. On the contrary, in the 3_03_←2_02_ transition (Figure 1), the deuterium-bonded dimer *t*-ACR-MeOD-1 shows a splitting of only ~0.16 MHz, while the hydrogen-bonded dimer *t*-ACR-MeOH-1 is split by ~0.18 MHz. The smaller A-E splittings in the deuterium-bound complexes are likely due to the Ubbelohde effect, which is the shortening of the HBs upon deuteration [47]. The effect of the shortening of the HB on the A-E splitting in deuterated complexes was also observed in the ethanol–methanol dimer [43]. The A-E splittings for the deuterated species *t*-ACR-MeOD-1 and *t*-ACR-MeOD-2 are summarized in Table S6.

In Table 2, the planar moments of inertia *M*_cc_ for the two species are also reported. Their values are consistent with an overall planar structure of the complex where only the methyl hydrogen atoms are out of the plane. The small differences between the values of the two conformers and those of the deuterated species with respect to the parent species can be attributed to small contributions of the low-frequency vibrational modes to the experimentally determined moments of inertia.

### 2.5. Possible Presence of Other Isomers

**Figure 3:**
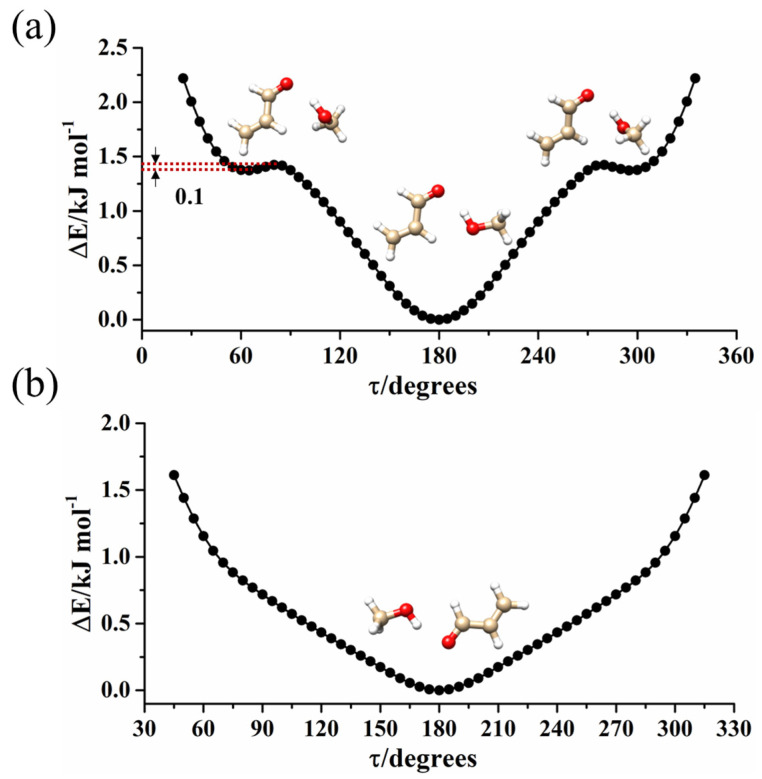
Ab initio (MP2/aug-cc-pVTZ) potential energy surface for the torsion of MeOH’s methyl group around the OH bond for the (**a**) *t*-ACR-MeOH-1and (**b**) *t*-ACR-MeOH-2. In the upper panel, this motion interconverts the in-plane conformer (absolute minimum) with the out-of-plane ones (relative equivalent minima).

Considering the relative energy value, the temperature prior to the expansion and the dipole moment component’s values, the most intense *μ*_a_ lines of *t*-ACR-MeOH-3, were predicted to be 72.0% of the most intense ones of *t*-ACR-MeOH-1. However, the spectrum originating from conformer *t*-ACR-MeOH-3 was not found in the experiment. This can be attributed to a relaxation of the population of this conformation onto that of the global minimum during adiabatic expansion, and in order to confirm this, the potential energy surface for the interconversion was calculated. This was performed by changing the dihedral angle *τ* = CO-HO on a regular grid with a step of 5° for both conformers, and the results are shown in Figure 3. In Figure 3a, one can see the conformations in which the MeOH oxygen is bound to the methylenic hydrogen atom (conformers *t*-ACR-MeOH-1 and *t*-ACR-MeOH-3), while in Figure 3b, are those where the MeOH oxygen is bound to the aldehydic hydrogen (conformer *t*-ACR-MeOH-2). In the first case, the higher energy conformer *t*-ACR-MeOH-3 gives rise to two equivalent minima, with respect to the ACR plane. The population’s relaxation of this conformation to the global minimum *t*-ACR-MeOH-1 during adiabatic expansion is demonstrated by the low barrier to interconversion between the two conformations (approximately 0.1 kJ mol^−1^). On the contrary, when the MeOH oxygen is bound to the aldehydic hydrogen (conformer *t*-ACR-MeOH-2), only one minimum is found, as shown in Figure 3b, and the non-planar arrangement does not correspond to a minimum. This can be ascribed to two reasons: the non-planar conformer exists but was not optimized due to the accuracy of the calculation method, or the non-planar conformer is not stable considering the weak intermolecular interaction between MeOH oxygen and aldehydic hydrogen.

### 2.6. Analysis and General Discussion of the Clustering Interaction

The structure and the PES shown in Figure 3a are similar to the ones observed in the acrylonitrile-MeOH complex [18] where the water molecular binds to acrylonitrile forming a cyclic planar structure with a OH···N HB and a CH···O_W_ (global minimum), while a non-planar structure stabilized by the same interactions, but with the methyl group perpendicular to the acrylonitrile plane, is at a relative minimum with two equivalent forms. Also, in that case, the barrier to interconversion was found to be quite low (about 0.6 kJ mol^−1^), preventing the observation of the non-planar conformer in the jet expansion.

**Figure 4:**
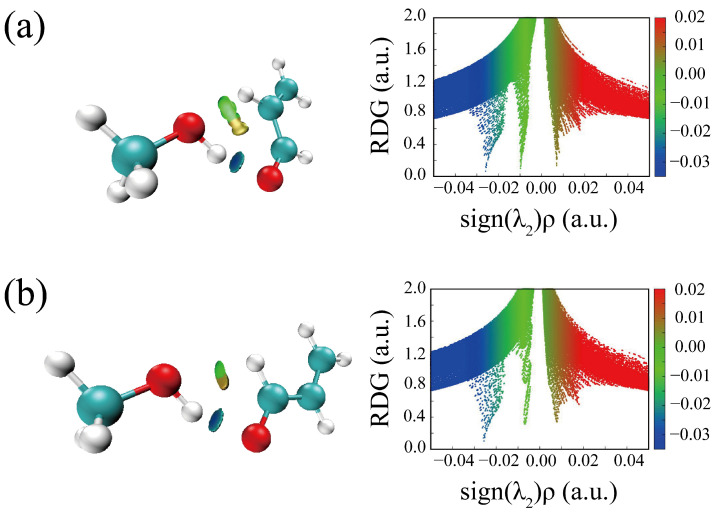

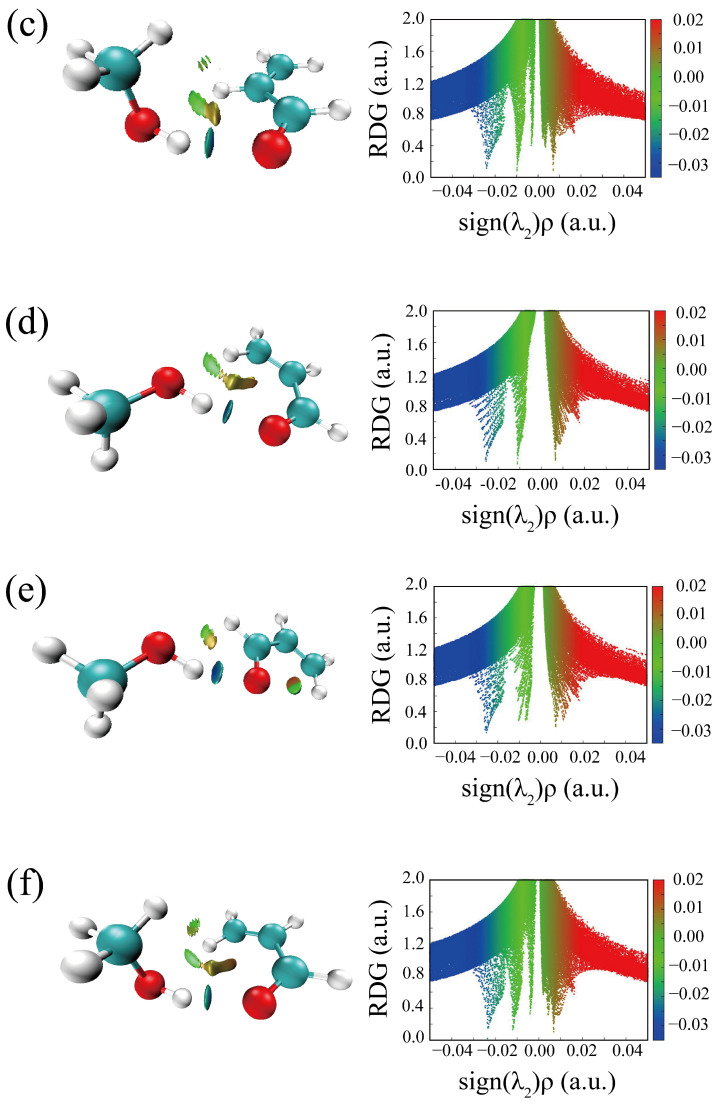
The NCI plots from the ab initio (MP2/aug-cc-pVTZ) outputs for conformers: (**a**) *t*-ACR-MeOH-1, (**b**) *t*-ACR-MeOH-2, (**c**) *t*-ACR-MeOH-3, (**d**) *c*-ACR-MeOH-1, (**e**) *c*-ACR-MeOH-2 and (**f**) *c*-ACR-MeOH-3. Left panel: gradient isosurfaces according to the values of the sign(λ_2_)ρ (−0.04~0.04 a.u.). Color coding is blue (stronger attractive interactions), green (weaker attractive interactions) and orange-red (repulsive interaction). Right panel: The reduced density gradient (RDG) versus sign(λ_2_)ρ. Positive values of the sign(λ_2_)ρ indicate repulsive interactions, and negative values of the sign(λ_2_)ρ indicate attractive interactions.

The HBs in the six optimized conformers can be better visualized by using Johnson’s NCI method [48]. Figure 4 displays the NCI plots, revealing the pattern of intermolecular interactions for all optimized conformations of ACR-MeOH. The gradient isosurfaces are colored according to the corresponding values of sign(λ_2_) ρ. A negative sign of sign(λ_2_)ρ (corresponding to blue regions) and small values of the reduced density gradient (RGD <0.5 a.u.) suggest stronger OH···O HB, while green regions indicate a weaker C–H···O attractive interaction. Positive values of sign(λ_2_)ρ and small values of the RGD (<0.5 a.u.), corresponding to orange regions, suggest a weak repulsive interaction. 

One can observe the relatively large orange regions between oxygen and hydrogen in the *c*-ACR frame (Figure 4d–f), suggesting a weak repulsive intramolecular interaction accounting for the lower stability of *c*-ACR compared to *t*-ACR where this repulsion is absent (Figure 4a–c). Focusing on the most stable conformations of each series, one can see that in conformations 1 (Figure 4a,d) and 2 (Figure 4b,e) the OH···O HB is of similar strength, while the CH···O interactions in conformations 1 (CCH···O_w_) are stronger than those in conformations 2 (O=CH···O_w_); this is in agreement with the results of the theoretical calculation where conformations 1 have lower energy than conformations 2. 

**Figure 5:**
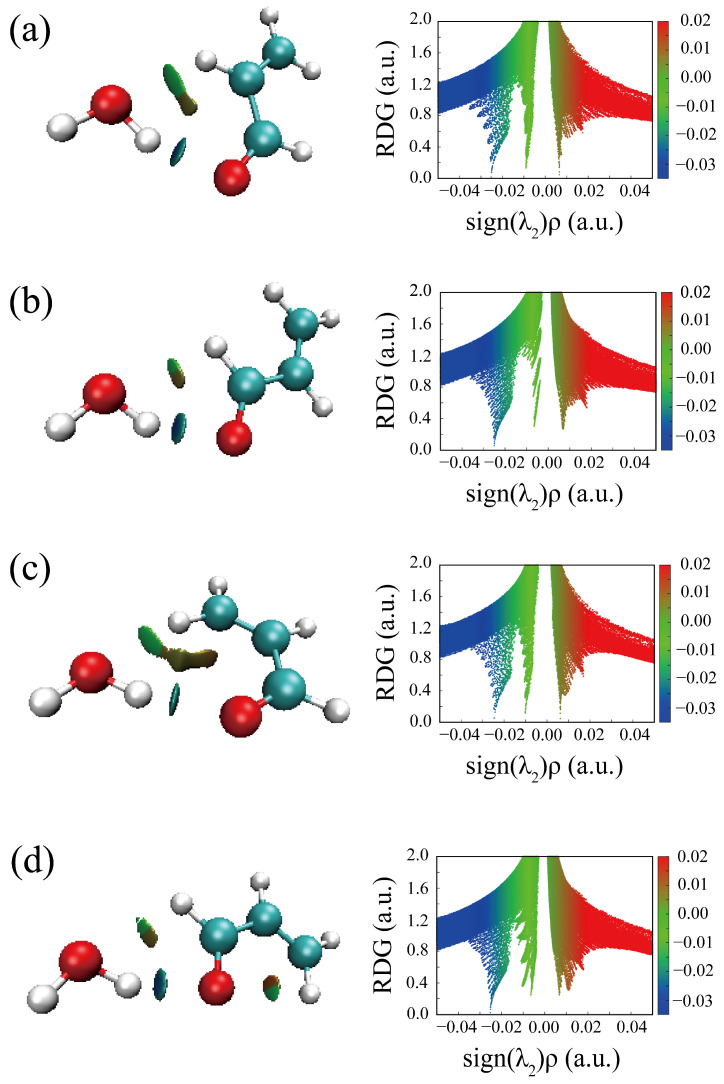
The NCI plots from the ab initio (MP2/aug-cc-pVTZ) outputs for three conformers: (**a**) *t*-ACR-W-1, (**b**) *t*-ACR-W-2, (**c**) *c*-ACR-W-1 and (**d**) *c*-ACR-W-2. (**Left panel**): gradient isosurfaces according to the values of the sign(λ_2_)ρ (−0.04~0.04 a.u.). Color coding is blue (stronger attractive interactions), green (weaker attractive interactions) and orange-red (repulsive interaction). (**Right panel**): The reduced density gradient (RDG) versus sign(λ_2_)ρ. Positive values of the sign(λ_2_)ρ indicate repulsive interactions, and negative values of the sign(λ_2_)ρ indicate attractive interactions.

For comparison, Johnson’s NCI method is also applied to the complexes of ACR-water, as shown in Figure 5. Two hydrogen bonds are formed between ACR and water in all four conformations (Figure 5a–d): a stronger OH···O HB (the blue regions) and a weaker CH···O interaction (the green regions), which correspond to the planar conformations of ACR-MeOH described above (Figure 4a,b,d,e), respectively. The strengths of hydrogen bonds are of the same level in view of the area of the NCI plots from ACR-water to ACR-MeOH. 

A careful analysis of the geometrical parameters can also be performed. The OH···O/CH···O distances are 1.914/2.353 Å and 1.922/2.608 Å in the ACR-MeOH conformers 1 and 2, respectively, and 1.923/2.385 Å and 1.928/2.636 Å in the ACR-W conformers 1 and 2. These values confirm the relative strength of the interactions discussed previously, with the OH···O being the stronger interaction compared to the CH···O one and the latter being strongest in conformer 1 compared to to conformer 2. 

Comparative analysis of the computational results of ACR-MeOH complexes and reported experimental geometries of MeOH and ACR reveals several key structural changes upon complexation. The O-H bond length in MeOH tends to be longer, and the ∠C5O6H11 angle is smaller after complexation. When compared to the ACR monomer, the complexation leads to a longer C3=O4 bond and an extended O4-H10 distance. Additionally, the ∠C2C3H10 angle becomes larger, whereas the angles ∠C1C2C3 and ∠O4=C3H10 decrease. For the ∠C2C3O4 angle, it tends to increase in *t*-ACR-MeOH-1 but decreases in *t*-ACR-MeOH-2. The determined barriers for the internal rotation of the methyl group in these complexes are lower than those found for the MeOH monomer. According to Fraser et al. [34], this is attributed to the effects of the librational motion of either the entire MeOH moiety or the methyl group within the complex. Schmitt [40] points out that the small absolute value of the barrier compared to the monomer is likely an artifact due to the one-dimensional description of the large amplitude motion. In MeOH-HCl complexes [38], the apparent barrier reduction arises from neglecting the large amplitude wagging motion of the hydroxyl hydrogen relative to HCl. In the acrylonitrile-MeOH complex [18], the results exclude the librational motion of the entire methanol moiety or the methyl group, but the motion of the hydroxyl group cannot be excluded. In summary, the decreased barrier is not due to complexation effects but is related to another large amplitude internal motion, such as the motion of the hydroxyl group coupled with the internal rotation of the methyl group.

Other complexes of small organic molecules with methanol have been characterized by rotational spectroscopy and, in all cases, the global minimum is stabilized by similar cyclic structures. In formamide-MeOH [41], there is an HB from the MeOH hydroxyl hydrogen to the oxygen atom of formamide and one from the N-H group of formamide to the MeOH oxygen. The corresponding OH···O and NH···O distances are quite short: 2.01(1) and 1.97(1) Å, respectively. In formaldehyde-MeOH [35], the HBs are from the MeOH hydroxyl hydrogen to the oxygen of formaldehyde (2.097(6) Å) and from a CH group of the second moiety to the MeOH oxygen, while in acrylonitrile-MeOH [18] the HB is from MeOH to the cyano group and from the methylenic hydrogen to the MeOH oxygen; the OH···N and CH···O distances being 2.257(1) and 2.484(1) Å (MP2/aug-cc-pVTZ values). The shorter distances in formamide-MeOH seem to imply a larger binding energy, which is indeed confirmed by the ab initio (MP2/aug-cc-pVTZ) value of 44.5 kJ mol^−1^ for this system compared to those calculated for formaldehyde-MeOH (24.1 kJ mol^−1^) and acrylonitrile-MeOH (26.3 kJ mol^−1^). For the ACR-MeOH system, the HB distances and energy binding values (30.4 kJ mol^−1^, MP2/aug-cc-pVTZ, Table 3) are found to be halfway between the three systems cited above. The HB structure for ACR-MeOH has a lower binding energy than formamide-MeOH and higher than formaldehyde-MeOH and acrylonitrile-MeOH. Notably, the binding energy value for ACR-MeOH is slightly larger than that of ACR−water (28.8 kJ mol^−1^ MP2/aug-cc-pVTZ, Table 3) [31].

A quantitative understanding of the chemical nature of the NCIs can be achieved by energy decomposition analysis, using the Symmetry-Adapted Perturbation theory (SAPT). According to SAPT [49], the energy of the intermolecular interaction can be interpreted as the sum of different terms with defined physical meanings: electrostatic, induction, dispersion and exchange-repulsion terms. The results, summarized in Table 3, show that the intermolecular interactions become slightly stronger following the substitution of the hydrogen atom in water with the methyl group in MeOH. These results are confirmed by the ab initio binding energies (MP2-aug-cc-pVTZ) and are also reported in Table 3. The dispersion interactions are mainly responsible for the increased total interactions and are related to electron correlations in the whole molecules, and they become stronger as the size of the molecules and the number of electrons increase. 

## 3. Methods

The experiment was carried out with the COBRA-type pulsed supersonic-jet FTMW spectrometer, which was previously described [50,51,52,53]. ACR and MeOH were acquired from Sigma-Aldrich (St. Louis, MO, USA) (purity > 99%), while D_2_O was acquired from Cambridge Isotope Laboratories, Inc. (Tewksbury, MA, USA) (purity 99.9%). All compounds were used without further purification. For the preparation of the complexes, samples of ACR (cooled to 273 K) and MeOH (at 298 K) were prepared in two separate containers, and helium, at a stagnation pressure of about 0.6 MPa, was made to flow over them creating a 1% mixture of both ACR and MeOH in the carrier gas. The hydroxyl-enriched deuterated isotope of methanol was prepared by directly mixing methanol with the D_2_O sample in a 1:2 volume ratio.

The molecular beam was expanded via a solenoid valve (General Valve, Series 9, nozzle diameter 0.5 mm) into a Fabry–Pérot cavity. This expansion allowed the molecules and their complexes to reach low rotational temperatures (1–2 K), trapping the most stable forms at their energy minimum. Spectral line positions were identified using Fourier transformation of the time-domain signal with 8 k data points recorded at a 100 ns sampling interval.

Geometry optimizations and subsequent harmonic frequency calculations were performed at the MP2/aug-cc-pVTZ level using the GAUSSIAN 16 program [54]. This method is known to provide very accurate results regarding both the geometries and energies. The small size of the system allowed the use of this method, which is more computationally expensive than DFT. The analysis of the spectral data was performed with the program XIAM [45], which is based on the combined axis method [55] and directly supplies the *V*_3_ barrier to internal rotation, the angles between the internal rotation axis and the principal axes and the moment of inertia of the internal top, while a set of rotational constants common to both the A- and E-states is provided (corresponding to the values for the infinite barrier limit). 

Johnson’s NCI method [48], which can visualize and quantify non-covalent interactions through the reduced density gradient (RDG) of the electron distribution, was used to study the non-covalent interactions between ACR and MeOH.

## 4. Conclusions

The rotational spectrum of the 1:1 complex of ACR and MeOH was studied to obtain information on its structure, on the HBs and on the barrier hindering the methyl group’s internal rotation in the complex. Two stable conformations of ACR-MeOH and their deuterated istopologues have been measured. The determined rotational constants are coherent with a cyclic structure (MP2/aug-cc-pVTZ) stabilized by a primary OH···O HB between MeOH and the aldehyde group and a secondary one between the terminal CH group and the MeOH oxygen, which were also characterized and visualized using NCI plots. Splitting of the rotational lines due to the hindered internal rotation of the methyl group was observed and the global analysis of the spectrum led to the determination of a *V*_3_ value of 2.686(3) kJ mol^−1^ and 2.723(6) kJ mol^−1^ for the two conformations, respectively. These values are about 40% lower than those experimentally determined for free MeOH, revealing a significant discrepancy between the theoretical and experimental rotational barriers. This demonstrates the importance of continuous improvement in computational chemistry techniques to achieve more accurate predictions and better alignment with experimental observations. The finding that the reduced mass of the internal rotation motion is extremely close to the calculated one allowed us to exclude that the lowering of the barrier could be ascribed to a librational motion of the methyl group. The present findings thus have implications for understanding large-amplitude motions within molecular complexes. Such insights provide valuable empirical evidence that can enhance the accuracy of theoretical models of internal dynamics in molecular chemistry, offering a deeper comprehension of the factors influencing molecular behavior.

## Figures and Tables

**Table 1:** Ab initio (MP2/aug-cc-pVTZ) structures, rotational constants, electric dipole moment components and relative energies of the different conformations of ACR-MeOH.

	*t*-ACR-MeOH-1	*t*-ACR-MeOH-2	*t*-ACR-MeOH-3
	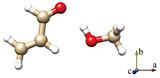	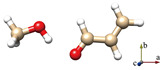	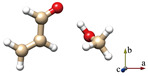
*A*, *B*, *C*/MHz *μ*_a_, *μ*_b_, *μ*_c_/D Δ*E*_e_/kJ mol^−1^ Δ*E*_0_/kJ mol^−1^	6233, 1390, 1145 −2.4, −0.5, 0.0 0 0	11158, 1036, 954 2.7, 0.6, 0.0 3.7 3.2	4905, 1692, 1373 −3.2, −1.4, 1.6 1.4 1.3
	***c*** **-ACR-MeOH-1**	***c*** **-ACR-MeOH-2**	***c*** **-ACR-MeOH-3**
	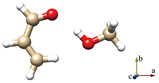	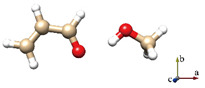	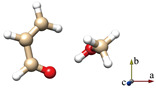
*A*, *B*, *C*/MHz *μ*_a_, *μ*_b_, *μ*_c_/D Δ*E*_e_/kJ mol^−1^ Δ*E*_0_/kJ mol^−1^	5974, 1505, 1211 −2.4, 0.0, 0.0 10.5 10.1	16485, 1007, 955 −2.5, 0.2, 0.0 13.5 12.7	4958, 1824, 1481 −3.0, −1.2, −1.8 11.0 10.9

**Table 2:** Experimental spectroscopic parameters of the parent and deuterated species of *t*-ACR-MeOH-1 and *t*-ACR-MeOH-2 and the deuterated species.

	*t*-ACR-MeOH-1	*t*-ACR-MeOD-1-	*t*-ACR-MeOH-2	*t*-ACR-MeOD-2-
*A*/MHz	6180.152(3) ^[a]^	6193.285(9)	11263.02(4)	11312(12)
*B*/MHz	1350.2493(7)	1345.401(2)	1009.515(3)	1004.652(2)
*C*/MHz	1116.9532(7)	1114.199(1)	933.289(3)	929.742(2)
*D*_J_/kHz	0.905(6)	0.905(6)	0.724(9)	0.71(1)
*D*_JK_/kHz	−4.30(9)	−4.7(1)	−37.1(2)	−38(1)
*d*_1/_kHz	−0.219(6)	−0.217(9)	−0.09(1)	−0.082(9)
*V*_3_/kJ mol^−1^	2.686(3)	2.825(3)	2.722(6)	2.856(1)
*δ*/deg	0.089(7)	0.09(2)	2.896(5)	2.881(4)
*F*_0_/GHz	158.6(1)	159.3(2)	156.1(3)	[156.1] ^[b]^
*Dc3J*/kHz	−1.25(5)	−1.4(1)	-	
*σ* ^[c]^/kHz	5.8	12.0	15.9	8.9
*N* ^[d]^	42	46	42	24
*M*_cc_/uÅ^2^	1.7991	1.8275	1.9915	2.0731

^[a]^ Error in parentheses in units of the last digit. ^[b]^ The data in brackets were fixed at the corresponding normal species values because they were not determined in the fit. ^[c]^ RMS error of the fit. ^[d]^ Number of lines in the fit.

**Table 3:** Ab initio binding energies calculated by MP2/aug-cc-pVTZ method (BE MP2), SAPT2+ (3)dMP2/aug-cc-pVTZ analysis and total binding energies (BE SAPT) for the complexes of methanol and water with acrolein (in bold the observed conformations), all values in kJ mol^−1^.

Energies	Electrostatic	Induction	Dispersion	Exchange	BE SAPT	BE MP2
**Acrolein-Methanol**						
***t*** **-ACR-** **MeOH** **-1**	−46.15	−16.16	−20.45	54.08	−28.68	−30.4
***t*** **-ACR-** **MeOH** **-** **2**	−38.67	−14.24	−17.15	44.22	−25.84	−26.8
*t*-ACR-MeOH-3	−43.84	−14.07	−22.36	53.60	−26.68	−29.1
*c*-ACR-MeOH-1	−45.38	−16.44	−20.75	54.23	−28.34	−29.4
*c*-ACR-MeOH-2	−38.39	−14.20	−17.04	44.08	−25.55	−26.3
*c*-ACR-MeOH-3	−43.96	−14.24	−23.41	54.59	−27.02	−28.9
**Acrolein-Water**						
***t*** **-ACR-** **W** **-1**	−44.43	−15.15	−17.96	49.78	−27.76	−28.8
***t*** **-ACR-** **W** **-** **2**	−37.85	−13.69	−15.27	41.47	−25.35	−25.8
*c*-ACR-W-1	−42.90	−14.94	−17.94	48.63	−27.14	−27.6
*c*-ACR-W-2	−37.57	−13.66	−15.17	41.34	−25.05	−25.4

## Data Availability

No new data were created or analyzed in this study.

## Supplementary Materials

The following supporting information can be downloaded at: https://www.mdpi.com/article/10.3390/molecules29153444/s1: Table S1: Experimental transition frequencies (ν/MHz) and observed minus calculated values (Δν/MHz) of t-ACR-MeOH-1; Table S2: Experimental transition frequencies (ν/MHz) and observed minus calculated values (Δν/MHz) of t-ACR-MeOH-2; Table S3: Experimental transition frequencies (ν/MHz) and observed minus calculated values (Δν/MHz) of t-ACR-MeOD-1; Table S4: Experimental transition frequencies (ν/MHz) and observed minus calculated values (Δν/MHz) of t-ACR-MeOD-2; Table S5: MP2/aug-cc-pVTZ geometries of the two conformers of the ACR-MeOH complexes; Table S6: The different splitting (ΔSplit/MHz) of A-E lines between parent species and deuterated species; Figure S1: MP2/aug-cc-pVTZ geometries of the two conformers of the ACR-MeOH complexes; Table S7: MP2/aug-cc-pVTZ geometries of the two conformers of the ACR-MeOH complexes and experimental geometries of MeOH and ACR; References [28,44].
