# Supplementary material for: Intermolecular Interactions between Aldehydes and Alcohols: Conformational Equilibrium and Rotational Spectra of Acrolein-Methanol Complex
Source: Molecules. 2024 Jul 23;29(15):3444. doi: 10.3390/molecules29153444 (PMC11313379; doi:10.3390/molecules29153444)
Supplement: Supplementary file 1 [file molecules-29-03444-s001.zip › molecules-3072903-supplementary.pdf]

Intermolecular Interactions between Aldehydes and Alcohols: Conformational Equilibrium and Rotational Spectra of Acrolein-Methanol Complex

Dingding Lv, [a] David Sundelin, [b] Assimo Maris, [a] Luca Evangelisti, [a] Wolf Dietrich Geppert, [b, *] Sonia Melandri [a, *]

[a] Dipartimento di Chimica "G. Ciamician" dell'Università, Via Selmi 2, I-40126 Bologna, Italy

b Fysikum, Stockholm University, Roslagstullsbacken 21, 106 91 Stockholm

*Corresponding authors: Prof. Sonia Melandri sonia.melandri@unibo.it; Prof. Wolf Geppert wgeppert@fysik.su.se.

KEYWORDS: acrolein, methanol, hydrogen bond, microwave spectroscopy; molecular structure; methyl internal rotation

ABSTRACT

The rotational spectra of the 1:1 complex formed by acrolein and methanol and its deuterated isotopologues have been analysed. Two stable conformations in which two hydrogen bonds between the two moieties are formed were detected. The rotational lines show a hyperfine structure due to the methyl group internal rotation in the complex and the V_3 barriers hindering the motion were determined as 2.629(5) kJ mol^{-1} and 2.722(5) kJ mol^{-1} for the two conformations, respectively. Quantum mechanical calculations at the MP2/aug-cc-pVTZ level and ccomprehensive analysis of the intermolecular interactions, utilizing NCI and SAPT approaches, highlight the driving forces of the interactions and allow the determination of the binding energies of complex formation.

INDEX

Table S1 Experimental transition frequencies (v/MHz) and observed minus calculated values (Δv/MHz) of *t*-ACR-MeOH-1.

Table S2 Experimental transition frequencies (v/MHz) and observed minus calculated values (Δv/MHz) of *t*-ACR-MeOH-2.

Table S3 Experimental transition frequencies (v/MHz) and observed minus calculated values (Δv/MHz) of *t*-ACR-MeOD-1.

Table S4 Experimental transition frequencies (v/MHz) and observed minus calculated values (Δv/MHz) of *t*-ACR-MeOD-2.

Table S5 MP2/aug-cc-pVTZ geometries of the two conformers of the ACR-MeOH complexes.

Table S6 The different splitting (Δ_{Split} / MHz) of A-E lines between parent species and deuterated species.

Figure S1 MP2/aug-cc-pVTZ geometries of the two conformers of the ACR-MeOH complexes.

Table S7 MP2/aug-cc-pVTZ geometries of the two conformers of the ACR-MeOH complexes and experimental geometries of MeOH and ACR.

References

Table S1 Experimental transition frequencies (v/MHz) and observed minus calculated values (Δv/MHz) of *t*-ACR-MeOH-1.

J'	K_a'	K_c'	J''	K_a''	K_c''	v_A	Δv_A	v_E	Δv_E	$\|\Delta_{A-E}\|$
3	1	3	2	1	2	7046.6260	0.0214	7068.3599	-0.0129	21.7339
1	1	1	0	0	0	7305.7175	-0.0352	7190.8750	0.02	114.8425
3	0	3	2	0	2	7368.6600	0.0002	7368.4800	-0.0076	0.18
3	2	2	2	2	1	7401.6220	0.0085	7417.7914	-0.0003	16.1694
3	2	1	2	2	0	7434.4795	0.0072	7418.2695	-0.0037	16.21
3	1	2	2	1	1	7746.1710	-0.0132	7724.2853	0.0007	21.8857
4	1	4	3	1	3	9386.2351	0.0253	9395.5371	-0.0176	9.302
2	1	2	1	0	1	9539.6625	-0.023	9480.9230	0.0151	58.7395
4	0	4	3	0	3	9786.8990	0.0075	9786.5630	-0.0047	0.336
4	2	3	3	2	2	9862.3325	0.0206	9899.3566	-0.0051	37.0241
4	2	2	3	2	1	9944.0027	-0.0039	9906.9896	-0.0101	37.0131
4	1	3	3	1	2	10318.0689	-0.0137	10308.5058	-0.0006	9.5631
3	1	3	2	0	2	11660.1396	-0.0005	11623.2166	0.0054	36.923
5	1	5	4	1	4	11718.4385	0.0321	11723.0429	-0.0253	4.6044
5	0	5	4	0	4	12174.0054	0.016	12173.4325	-0.0105	0.5729
5	1	4	4	1	3	12880.7055	-0.0129	12875.6853	0.0059	5.0202
4	1	4	3	0	3	13677.7065	0.0164	13650.2659	-0.0124	27.4406
6	1	6	5	1	5	14042.0113	0.0313	14044.4902	-0.0325	2.4789
6	0	6	5	0	5	14525.1437	0.0232	14524.2604	-0.0237	0.8833
6	1	5	5	1	4	15431.0750	-0.0062	15427.9723	0.0123	3.1027
5	1	5	4	0	4	15609.2463	0.0414	15586.7227	-0.0561	22.5236

Table S2 Experimental transition frequencies (v/MHz) and observed minus calculated values (Δv/MHz) of *t*-ACR-MeOH-2.

J'	K_a'	K_c'	J''	K_a''	K_c''	v_A	Δv_A	v_E	Δv_E	$\lvert\Delta_{A-E}\rvert$
4	1	4	3	1	3	7618.1652	0.0004	7669.5224	-0.0693	51.3572
4	0	4	3	0	3	7766.8648	0.0169	7766.7462	-0.0272	0.1186
4	2	3	3	2	2	7771.9530	0.0289	7774.0058	-0.0243	2.0528
4	2	2	3	2	1	7776.1669	0.0265	7773.9603	-0.0014	2.2066
4	1	3	3	1	2	7922.9116	0.041	7871.4050	0.052	51.5066
5	1	5	4	1	4	9521.8170	0.0208	9557.8450	-0.0727	36.0280
5	0	5	4	0	4	9705.2723	0.0008	9705.1613	-0.0054	0.1110
5	2	4	4	2	3	9714.2845	0.0354	9718.3899	-0.074	4.1054
5	2	3	4	2	2	9722.7046	0.0258	9718.3356	-0.0462	4.3690
5	1	4	4	1	3	9902.6991	0.0403	9866.4500	0.0328	36.2491
6	1	6	5	1	5	11424.8810	0.0517	11448.9900	-0.039	24.1090
6	0	6	5	0	5	11641.5344	0.0258	11641.3295	-0.0361	0.2049
6	2	5	5	2	4	11656.1771	0.0402	11663.4286	-0.036	7.2515
6	2	4	5	2	3	11670.9086	0.0282	11663.4568	-0.01	7.4518
6	1	5	5	1	4	11881.8252	0.0058	11857.4600	-0.006	24.3652
1	1	1	0	0	0	12224.8994	0.0266	11878.5434	0.0008	346.3560
7	1	7	6	1	6	13327.2525	0.1002	13343.4699	0.0034	16.2174
7	0	7	6	0	6	13575.1642	0.0326	13574.8904	-0.0505	0.2738
7	1	6	6	1	5	13860.1917	-0.0273	13843.6488	-0.0633	16.5429
2	1	2	1	0	1	14091.6479	0.0134	13804.9290	0.0088	286.7189
3	1	3	2	0	2	15920.5022	-0.0034	15697.0406	-0.0453	223.4616

Table S3 Experimental transition frequencies (v/MHz) and observed minus calculated values (Δv/MHz) of *t*-ACR-MeOD-1.

J'	K_a'	K_c'	J''	K_a''	K_c''	v_A	Δv_A	v_E	Δv_E	$\|\Delta_{A-E}\|$
3	1	3	2	1	2	7027.0586	0.0172	7043.4129	-0.0443	16.3543
1	1	1	0	0	0	7314.8287	0.0446	7223.9852	-0.0378	90.8435
3	0	3	2	0	2	7346.5420	0.0002	7346.3864	0.0086	0.1556
3	2	2	2	2	1	7378.8134	-0.0048	7394.5184	0.0012	15.7050
3	2	1	2	2	0	7411.0021	0.0057	7395.2325	-0.0026	15.7696
3	1	2	2	1	1	7720.3381	-0.024	7703.8324	0.0333	16.5057
4	1	4	3	1	3	9360.3391	0.026	9367.1896	-0.0253	6.8505
2	1	2	1	0	1	9543.2571	0.0512	9498.1757	-0.0414	45.0814
4	0	4	3	0	3	9758.1844	0.0035	9757.8938	0.0032	0.2906
4	2	3	3	2	2	9832.0584	0.0076	9867.5611	0.0026	35.5027
4	2	2	3	2	1	9912.0485	-0.0151	9876.5437	0.0156	35.5048
4	1	3	3	1	2	10283.8366	-0.0329	10276.7442	0.0188	7.0924
3	1	3	2	0	2	11659.2082	0.0807	11630.5405	-0.0973	28.6677
5	1	5	4	1	4	11686.3611	0.041	11689.7052	-0.0235	3.3441
5	0	5	4	0	4	12139.3056	0.0123	12138.8156	-0.0064	0.4900
5	1	4	4	1	3	12838.2829	-0.0345	12834.5547	0.0206	3.7282
6	1	6	5	1	5	14003.8979	0.0336	14005.6764	-0.0239	1.7785
6	1	5	5	1	4	15380.7339	-0.0347	15378.4092	0.0282	2.3247
7	1	7	6	1	6	16312.1489	0.033	16313.0716	-0.0451	0.9227
7	0	7	6	0	6	16793.8495	0.0316	16792.8117	-0.0343	1.0378
7	2	6	6	2	5	17153.7396	0.0532	17203.8502	-0.0512	50.1106
7	2	5	6	2	4	17575.4496	-0.0869	17525.6552	0.0834	49.7944
7	1	6	6	1	5	17907.8176	-0.0254	17906.0997	0.0373	1.7179

Table S4 Experimental transition frequencies (v/MHz) and observed minus calculated values (Δv/MHz) of *t*-ACR-MeOD-2.

J'	K_a'	K_c'	J''	K_a''	K_c''	v_A	Δv_A	v_E	Δv_E	$\|\Delta_{A-E}\|$
4	1	4	3	1	3	7587.1949	0.0103	7629.6822	0.083	42.4873
4	0	4	3	0	3	7733.3951	-0.1417	7733.287	-0.0971	0.1081
4	1	3	3	1	2	7886.6855	0.0766	7844.0473	0.0691	42.6382
5	1	5	4	1	4	9483.1378	0.0067	9511.3792	0.0619	28.2414
5	0	5	4	0	4	9663.5796	-0.1213	9663.4362	-0.0669	0.1434
5	1	4	4	1	3	9857.4421	0.0506	9828.9955	0.066	28.4466
6	1	6	5	1	5	11378.5189	-0.0003	11396.7915	0.0254	18.2726
6	0	6	5	0	5	11591.6608	-0.0629	11591.4774	0.0013	0.1834
6	1	5	5	1	4	11827.5806	-0.0058	11809.0461	0.0461	18.5345
7	1	7	6	1	6	13273.2171	-0.0281	13285.2877	-0.0101	12.0706
7	0	7	6	0	6	13517.2298	0.0437	13516.9923	0.1091	0.2375
7	1	6	6	1	5	13796.9695	-0.098	13784.5897	-0.0179	12.3798

Table S5 MP2/aug-cc-pVTZ geometries of the two conformers of the ACR-MeOH complexes.

Bond lengths / Å		Valence angles/ °		Dihedral angles / °	
***t*-ACR-MeOH-1**					
C2C1	1.340				
C2C2	1.465	C2C2C1	118.9		
O4C3	1.224	O4C3C2	124.7	O4C3C2C1	180.0
C5O4	3.787	C5O4C3	123.3	C5O4C3C2	0.0
O6C5	1.420	O6C5O4	39.9	O6C5O4C3	-0.1
H7C1	1.080	H7C1C2	121.9	H7C1C2C3	-180.0
H8C1	1.084	H8C1C2	120.5	H8C1C2C3	0.0
H9C2	1.083	H9C2C1	123.1	H9C2C1C3	180.0
H10C3	1.104	H10C3C2	115.3	H10C3C2C1	0.0
H11O6	0.970	H11O6C5	108.5	H11O6C5O4	0.0
H12C5	1.087	H12C5O4	147.1	H12C5O4C3	-0.2
H13C5	1.092	H13C5O4	89.9	H13C5O4C3	125.4
H14C5	1.092	H14C5O4	89.9	H14C504C3	-125.7
***t*-ACR-MeOH-2**					
C2C1	1.339				
C2C2	1.464	C2C2C1	119.7		
O4C3	1.225	O4C3C2	123.8	O4C3C2C1	-180.0
O5O4	2.827	O5O4C3	90.7	O5O4C3C2	179.9
C6O5	1.419	C6O5O4	125.4	C6O5O4C3	180.0
H7C1	1.080	H7C1C2	122.0	H7C1C2C3	180.0
H8C1	1.083	H8C1C2	120.3	H8C1C2C3	0.0
H9C2	1.082	H9C2C1	122.7	H9C2C1C3	-180.0
H10C3	1.103	H10C3C2	115.9	H10C3C2C1	0.0
H11O5	0.970	H11O5O4	17.1	H11O5O4C3	180.0
H12C6	1.087	H12C6O5	107.2	H12C6O5O4	-179.9
H13C6	1.092	H13C6O5	111.9	H13C6O5O4	-61.1
H14C6	1.092	H14C6O5	111.9	H14C6O5O4	61.3

Table S6 The different splitting (Δ_{Split} / MHz) of A-E lines between parent species and deuterated species.

J'	K_a'	K_c'	J''	K_a''	K_c''	$\|\Delta_{A-E}\|$		Δ_{Split}
						***t*-ACR-MeOH-1**	***t*-ACR-MeOD-1**	
3	1	3	2	1	2	21.7339	16.3543	-5.3796
1	1	1	0	0	0	114.8425	90.8435	-23.999
3	0	3	2	0	2	0.18	0.1556	-0.0244
3	2	2	2	2	1	16.1694	15.705	-0.4644
3	2	1	2	2	0	16.21	15.7696	-0.4404
3	1	2	2	1	1	21.8857	16.5057	-5.38
4	1	4	3	1	3	9.302	6.8505	-2.4515
2	1	2	1	0	1	58.7395	45.0814	-13.6581
4	0	4	3	0	3	0.336	0.2906	-0.0454
4	2	3	3	2	2	37.0241	35.5027	-1.5214
4	2	2	3	2	1	37.0131	35.5048	-1.5083
4	1	3	3	1	2	9.5631	7.0924	-2.4707
3	1	3	2	0	2	36.923	28.6677	-8.2553
5	1	5	4	1	4	4.6044	3.3441	-1.2603
5	0	5	4	0	4	0.5729	0.49	-0.0829
5	1	4	4	1	3	5.0202	3.7282	-1.292
						***t*-ACR-MeOH-2**	***t*-ACR-MeOD-2**	
4	1	4	3	1	3	51.3572	42.4873	-8.8699
4	0	4	3	0	3	0.1186	0.1081	-0.0105
4	1	3	3	1	2	51.5066	42.6382	-8.8684
5	1	5	4	1	4	36.028	28.2414	-7.7866
5	0	5	4	0	4	0.111	0.1434	0.0324
5	1	4	4	1	3	36.2491	28.4466	-7.8025
6	1	6	5	1	5	24.109	18.2726	-5.8364
6	0	6	5	0	5	0.2049	0.1834	-0.0215
6	1	5	5	1	4	24.3652	18.5345	-5.8307
7	1	7	6	1	6	16.2174	12.0706	-4.1468
7	0	7	6	0	6	0.2738	0.2375	-0.0363
7	1	6	6	1	5	16.5429	12.3798	-4.1631

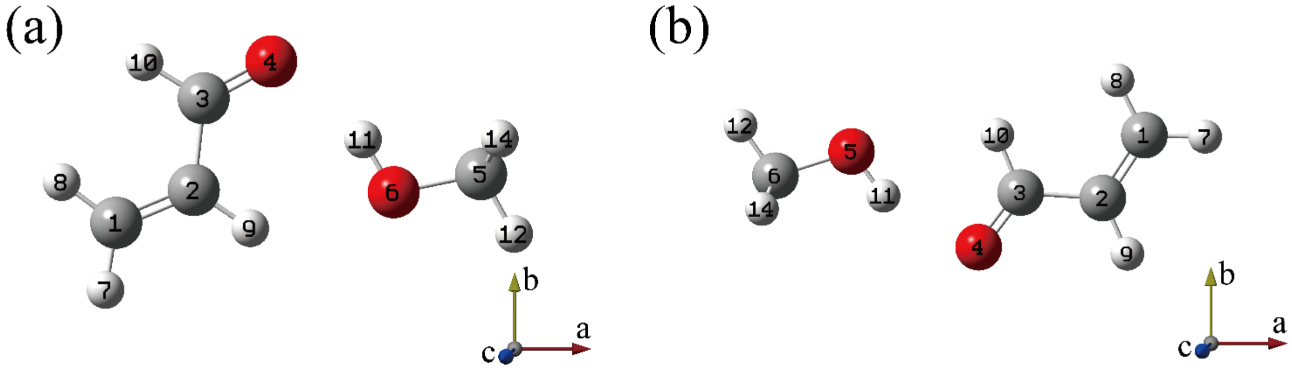

Figure S1 MP2/aug-cc-pVTZ geometries of the two conformers of the ACR-MeOH complexes.

Table S7 MP2/aug-cc-pVTZ geometries of the two conformers of the ACR-MeOH complexes and experimental geometries of MeOH and ACR.

Structure	**MeOH** [1]	*t*-ACR-MeOH-1	*t*-ACR-MeOH-2
r(O5H11) / Å	0.945(3)	0.9703	0.9696
∠C6O5H11 / °	108.53(5)	108.50	108.27
	***t*-ACR** [2]		
r(C3=O4) / Å	1.214(4)	1.2242	1.2246
r(O4H10) / Å	2.0149	2.0179	2.0200
∠C2C3=O4 / °	124.0(6)	124.66	123.77
∠C1C2C3 / °	120.4(5)	118.95	119.69
∠C2C3H10 / °	114.7(5)	115.28	115.93
∠O4=C3H10 / °	121.3(6)	120.06	120.31

References

1. Lees, R.M.; Lovas, F.J.; Kirchhoff, W.H.; Johnson, Dr. Microwave Spectra of Molecules of Astrophysical Interest: III. Methanol. *J Phys Chem Ref Data* **1973**, *2*, 205–214.
2. Blom, C.E.; Grassi, G.; Bauder, A. Molecular Structure of S-Cis-and s-Trans-Acrolein Determined by Microwave Spectroscopy. *J Am Chem Soc* **1984**, *106*, 7427–7431.
